# Genetics of Clubroot and Fusarium Wilt Disease Resistance in Brassica Vegetables: The Application of Marker Assisted Breeding for Disease Resistance

**DOI:** 10.3390/plants9060726

**Published:** 2020-06-09

**Authors:** Hasan Mehraj, Ayasha Akter, Naomi Miyaji, Junji Miyazaki, Daniel J. Shea, Ryo Fujimoto, Md. Asad-ud Doullah

**Affiliations:** 1 Graduate School of Agricultural Science, Kobe University, Rokkodai, Nada-ku, Kobe 657-8501, Japan or aakterhort@bau.edu.bd (A.A.); 162a318a@stu.kobe-u.ac.jp (N.M.); leo@people.kobe-u.ac.jp (R.F.); 2 Department of Horticulture, Bangladesh Agricultural University, Mymensing 2202, Bangladesh; 3 Agriculture Victoria Research Division, Department of Jobs, Precincts and Regions, AgriBioscience, Bundoora, VIC 3083, Australia; junji.miyazaki@agriculture.vic.gov.au; 4 Iwate Biotechnology Research Center, Narita, Kitakami, Iwate 024-0003, Japan; dshea30@bloomberg.net; 5 Department of Plant Pathology and Seed Science, Faculty of Agriculture, Sylhet Agricultural University, Sylhet 3100, Bangladesh

**Keywords:** clubroot, Fusarium wilt, *R* gene, quantitative trait locus, marker-assisted selection, Brassica

## Abstract

The genus Brassica contains important vegetable crops, which serve as a source of oil seed, condiments, and forages. However, their production is hampered by various diseases such as clubroot and Fusarium wilt, especially in Brassica vegetables. Soil-borne diseases are difficult to manage by traditional methods. Host resistance is an important tool for minimizing disease and many types of resistance (*R*) genes have been identified. More than 20 major clubroot (CR) disease-related loci have been identified in Brassica vegetables and several CR-resistant genes have been isolated by map-based cloning. Fusarium wilt resistant genes in Brassica vegetables have also been isolated. These isolated *R* genes encode the toll-interleukin-1 receptor/nucleotide-binding site/leucine-rice-repeat (TIR-NBS-LRR) protein. DNA markers that are linked with disease resistance allele have been successfully applied to improve disease resistance through marker-assisted selection (MAS). In this review, we focused on the recent status of identifying clubroot and Fusarium wilt *R* genes and the feasibility of using MAS for developing disease resistance cultivars in Brassica vegetables.

## 1. Introduction

The genus Brassica belongs to the family Brassicaceae (Cruciferae) containing 37 different species (http://www.theplantlist.org) and has great economic importance [1]. Three species, *Brassica rapa* L. (2n = 20, AA) and *Brassica oleracea* L. (2n = 18, CC) and its allotetraploid species, *Brassica napus* L. (2n = 38, AACC) are included in the genus Brassica and comprise commercially important vegetable and oilseed crops. *B. rapa* includes leafy vegetables such as Chinese cabbage (var. *pekinensis*), pak choi (var. *chinensis*), and komatsuna (var. *perviridis*), root vegetables such as turnip (var. *rapa*), and oilseed (var. *oleifera*). *B. oleracea* comprises commercially important vegetable crops with morphological variations such as cabbage (var. *capitata*), broccoli (var. *italica*), kale (var. *acephala*), kohlrabi (var. *gongylodes*), and cauliflower (var. *botrytis*). *B. napus* includes the oilseed crop, canola/rapeseed.

Various pathogens such as clubroot, Fusarium wilt, black rot, Sclerotinia stem rot, blackleg, white rust, downy mildew, white leaf spot, and turnip mosaic virus can infect Brassica crops [2,3]. Cultural, physical, biological, or chemical controls, or a combination of these controls, integrated pest management, are used for disease control. If plants have natural resistance against these pathogens, the dependence on these controls is reduced and is cost-effective. Thus, disease resistance is an important trait in plant breeding to prevent quality and yield losses.

The first tier of plant immunity is called pathogen-associated molecular pattern (PAMP)-triggered immunity (PTI) [4,5]. Plants recognize pathogens through the PAMPs by pattern recognition receptors (PRRs) [6] and this recognition leads to the activation of PTI. PTI induces the expression of defense genes such as the mitogen-associated protein kinase (MAPK) cascade or WRKY transcription factors [7,8]. In contrast, pathogens deliver virulence molecules called as effectors to suppress PTI [4]. The failure of PTI defense helps to activate an immune response called effector-triggered immunity (ETI), when plants recognize the effectors (Avr proteins) through disease resistance (R) proteins, an ETI is activated [5]. This recognition between R and Avr is termed ‘gene-for-gene resistance’ [9]. ETI is stronger against newly adapted pathogens in host plants than PTI [10]. R proteins contain nucleotide-binding (NB) and leucine rich repeat (LRR) domains, which are called NBS-LRR (nucleotide-binding site leucine-rich repeat) protein. NBS-LRR proteins are separated into two types by their N-terminus domain, either having a toll interleukin-1 receptor (TIR) domain (TIR-NBS-LRR protein) or coiled-coil (CC) domains (CC-NBS-LRR protein) [11,12,13]. In general, the LRR domain provides recognition specificity, the NB domain regulates activation, and the TIR domain regulates downstream signaling [5]. Besides this, some *R* genes also encode transmembrane receptor-like protein (RLPs), transmembrane receptor-like kinases (RLKs), cytoplasmic kinases (CKs), and proteins with atypical molecular motifs [4]. The constitution of *R* genes is different between monocotyledonous and dicotyledonous genomes. TIR-NBS-LRR genes are mostly absent in monocotyledons, while TIR-NBS-LRR genes are present in dicotyledons and usually more abundant than CC-NBS-LRR genes [13]. The R genes have been comprehensively identified in several species of the genus Brassica [14,15,16].

In a practical sense, the successful deployment of a novel *R* gene into a crop depends on the identification of a positive phenotype, the identification of genetic markers for marker-assisted selection (MAS) breeding, and understanding of how the novel resistance will behave under different genetic backgrounds and pathogenic pressures in the field. Clubroot and Fusarium wilt are considered as devastating diseases, and they cause a significant yield loss of Brassica vegetables for many years over the world. Some clubroot-resistant lines are susceptible to the Fusarium wilt and vice versa. In this review, we focus on recent knowledge about *R* genes of clubroot and Fusarium wilt as several important *R* genes/quantitative trait loci (QTL) against these pathogens have been identified in Brassica vegetables. In addition, MAS has been used to improve the disease resistance, and several cultivars with higher resistance in Brassica vegetables have recently been developed. We will introduce recent information about *R* genes and the prospect of their possible utilization for Brassica breeding.

## 2. Infection Process of the Pathogens

### 2.1. Infection Process of Clubroot Pathogen P. brassicae

**Figure 1:**
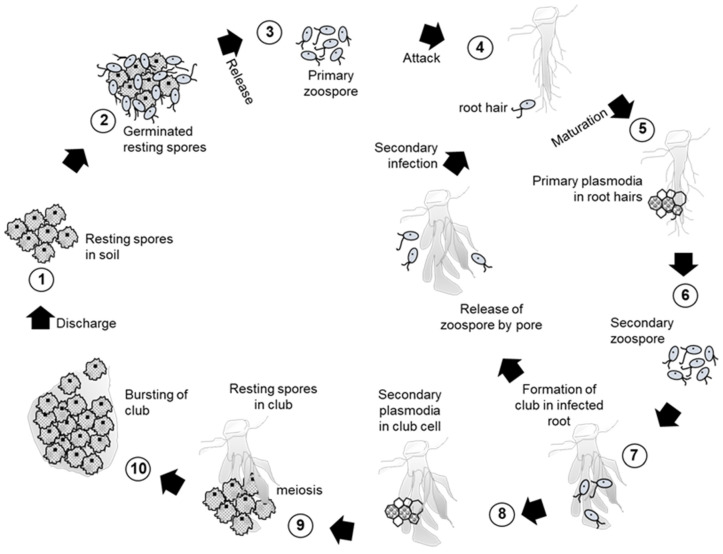
Infection process of clubroot disease caused by *Plasmodiophora brassicae*.

Clubroot is caused by the obligate parasite *Plasmodiophora brassicae* Woronin and is recognized as a major devastating disease in Brassicaceae that poses an emerging threat to Brassica crop production [17]. Clubroot disease was first reported in Russia in 1878 by Woronin and rapidly expanded to other countries like Europe, Brazil, South Africa, Australia, New Zealand, and China [17]. The infection of plants by *P. brassicae* is a two-phase process (Figure 1). The resting spores in soil germinate and the resultant zoospores then attack the plant’s root hairs. The zoospores then grow into multi-nucleate plasmodia (primary plasmodia) within the root hairs. The plasmodia cleave the root tissues and form secondary zoospores. The secondary zoospores penetrate into the root cortical tissues in a process known as cortical infection [18,19]. This cortical infection induces abnormal growth by the development of secondary plasmodia inside the affected cell, and proliferation of the secondary plasmodia leads to the formation of distorted massive gall known as club [18,19,20]. During the development of the pathogen in the plant, these secondary zoospores are capable of infecting the same plant or adjacent plants, thus repeating the cycle. Secondary plasmodia develop into multinuclear plasmodia by a number of nuclear divisions, and further meiosis may appear before the formation of numerous resting spores within the diseased plant tissue [19,20]. Resting spores are released into soil by the decay of clubs and survive for many years in soil. The spores are spread field-to-field via drainage water and infected root debris. Clubroot inhibits nutrient and water transport, resulting in wilting and ultimately the death of the infected plant. It is difficult to control clubroot infection by any means except genetic resistance cultivars due to the longevity of the resting spores. Crop rotation by clubroot resistant cultivars can reduce 100% of the clubroot severity compared with the susceptible cultivars [21]. Practicing two or more years of crop rotation by clubroot resistant cultivars with clubroot host significantly reduces the resting spores in soil, which is near to complete eradication of clubroot [22]. The effective and sustainable clubroot management by clubroot resistant cultivars is now disclosed, and hints at the importance of resistant cultivars for clubroot management. On the other hand, the host-range of the pathogen is mostly restricted within Brassicaceae species [19,23,24].

### 2.2. Infection Process of Fusarium Wilt Pathogen Foc/For

Yellowing or Fusarium wilt is caused by *Fusarium oxysporum f. sp. conglutinans*/*rapae* (*Foc*/*For*). Fusarium wilt disease was first reported in the USA, then in Japan and China, and has now been found almost all over the world [25,26]. The pathogen (*Foc*, *Fusarium oxysporum f. sp. conglutinans)*/*For*, *Fusarium oxysporum f. sp. rapae*) usually invades plants through their young roots, but can also invade through wounds in older roots [27,28]. This pathogen moves into and colonizes the xylem tissues, blocking vascular transport, leading to leaf yellowing, wilting, and defoliation, and in older plants, stunting and plant death [29,30]. The browning of vascular tissues can be observed in the stem and petiole of late-stage infected plants. It is a warm-weather disease and is active between 16 °C and 35 °C. The disease is more severe in warm conditions (above 24 °C) and not a problem in cool conditions [28,29]. The pathogen can survive in soil, seeds, and seedlings and can spread through water such as rain and flood [27,28] and remain for several years as resting spores in the soil. Two *forma specialis* (f. sp.) of *F. oxysporum* can cause disease in Brassicaceae. *Foc* causes disease in *B. oleracea* and *B. rapa* and *For* is specific to *B. rapa* [31]. Only two races in the *Foc*, race 1 and race 2, have been reported in the genus Brassica to date: race1 has been found worldwide and race 2 has only been found in USA and Russia [32].

## 3. Identification and Molecular Mechanism of Clubroot Resistant (CR) Genes

### 3.1. CR Loci in B. rapa

**Figure 2:**
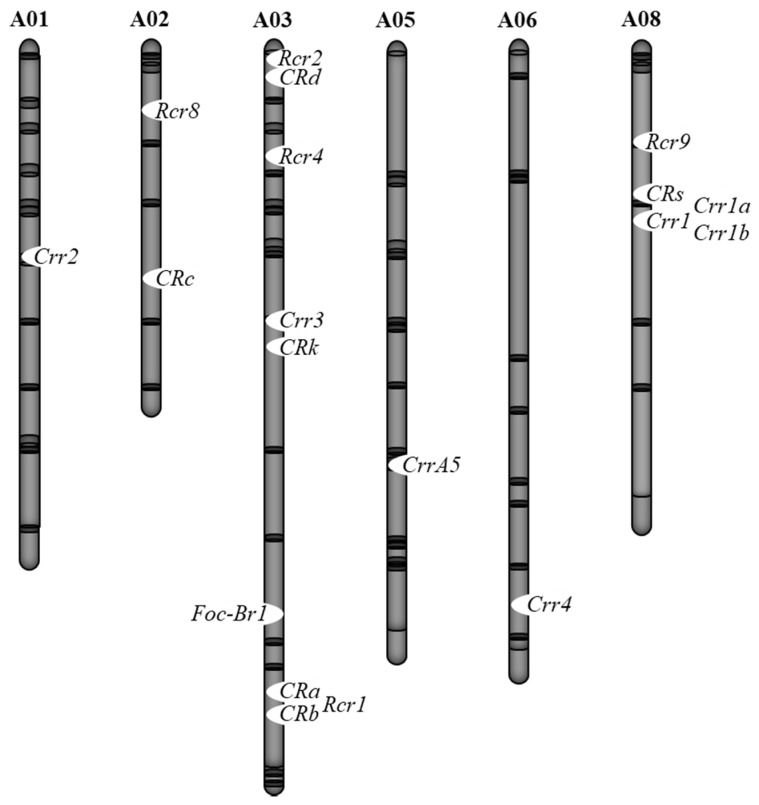
Chromosomal locations of clubroot resistant (CR) and Fusarium wilt resistant loci in *B. rapa*.

Clubroot disease resistance has been extensively studies in the genus Brassica. Several *CR* genes have been identified and mapped in *B. rapa*, *B. oleracea*, and other Brassica species [33]. In *B. rapa*, 18 major *CR* loci have been identified (Figure 2, Table 1); *Crr2* mapped on chromosome 1 [34], *CRc* and CR QTL, designated as *Rcr8*, on chromosome 2 [35,36], *Crr3*, *CRa*, *CRb*, *CRd*, *CRk*, *Rcr1*, *Rcr2*, and *Rcr4* on chromosome 3 [35,36,37,38,39,40,41,42,43,44,45,46,47,48,49,50], *CrrA5* on chromosome 5 [51], *Crr4* on chromosome 6 [52], *Crr1* (*Crr1a*, *Crr1b*), *Rcr9*, and *CRs* on chromosome 8 [36,44,53,54]. Most of the *CR* genes were identified through QTL mapping using a range of resistant sources based on molecular markers, genotyping-by-sequencing (GBS), or bulked segregant RNA sequencing (BSR-seq) strategies.

The first *CR* gene was identified in the turnip cultivar Siloga using a doubled haploid (DH) population [55] and a dominant major gene *CRa* was mapped on chromosome 3. A candidate gene of *CRa* has been identified, and it encodes a TIR-NBS-LRR protein [37]. *Crr1a* and *CRb* genes have also been identified by map-based cloning [38,46,53]. *CRb* was isolated independently of *CRa*, but they were identical genes [37,38]. *Crr1a* encodes TIR-NBS-LRR [38]. 

Recently, proteomics in Chinese cabbage during response to *P. brassicae* infection identified differentially expressed proteins (DEPs) between the susceptible and resistant lines [56]. Gene ontology analysis using DEPs showed that the category of ‘Glutathione transferase activity’ was overrepresented, suggesting that glutathione transferase is responsible for protecting plants from disease [56].

### 3.2. CR Loci in B. oleracea 

In contrast to *B. rapa*, no major *CR* genes or lines with strong resistance have been identified in *B. oleracea* [57]; only a few completely resistant accessions have been identified in *B. oleracea*. Genetic analysis of *CR* in *B. oleracea* was studied using diallel crossing methods or segregating populations. Only one major resistance gene, *Rcr7,* has been identified, and it might be located on chromosome 7 (LG 7) in cultivars, Tekila and Kilaherb of cabbage [57]. About fifty QTLs have been identified in the studies using different populations of *B. oleracea* (Table 2): three QTLs in broccoli [58], two in kale [59], two in cabbage [60], one in kale [61], three in kale [62], nine in kale [63], five in cabbage [64], three in cabbage using the GBS technique [65], and twenty-three QTLs in cabbage using single-nucleotide polymorphism (SNP) microarray technique [66]. The identification of several *CR* loci indicates that clubroot resistance in *B. oleracea* is controlled in a polygenic manner, confirming the complex genetic basis of the resistance, where a single resistance locus is not enough to confer sufficient resistance [67]. The comparison of these QTLs is currently impossible due to a lack of common molecular markers among different researchers and the use of different *CR* sources and races of pathogen [64].

### 3.3. CR Loci other Brassica Species

In *B. napus*, the majority of CR identified genes are derived from *B. rapa* var. *rapifera* [57]. In *B. napus*, one dominant gene and more than 30 QTLs were identified (Table 3). Two QTLs, CR2a and CR2b, were identified using Rutabaga (cv. Wilhelmsburger) showing resistance to race 2 of *P. brassicae* [68]. A major gene, *Pb-Bn1*, mapped on chromosome A03 was reported first and two minor QTLs were mapped on linkage groups C02 and C09 [69]. Nineteen race-specific resistance QTLs were mapped on eight different chromosomes, including the A genome (A02, A03, A08, A09) and C genome (C03, C05, C06, C09) [70]. Besides this, five QTLs using a DH line of canola against pathotype 3 [71], and nine QTLs from different accession of oilseed rape were identified, seven of which were novel through integrative analysis [8]. They first applied genome-wide association study (GWAS) based on whole-genome SNP data to detect that nine QTLs and reported that these QTLs cover genes encoding TIR-NBS gene family [8]. Some resistance loci with one dominant and two recessive loci were identified [72], and one locus linked to *CRa* gene [73] and a genomic region on chromosome A08 carrying resistance to all five pathotypes, namely pathotypes 2, 3, 5, 6, and 8, were also identified [74]. This suggests that a single gene or a cluster of genes located in this genomic region is involved in the control of resistance to these pathotypes [74]. Recently, two major loci on chromosome A02 and A03 controlling resistance, and seven minor loci, were identified by a SNP association analysis [75].

A single dominant gene *Rcr6* was also identified on chromosome 3 of the B genome (B03) through BSR-Seq and further mapped with Kompetitive Allele Specific PCR (KASP) analysis in *Brassica nigra* lines PI 219,576 [33]. The authors declared that *Rcr6* was the first gene identified and mapped in the B genome of Brassica species. All of the *CR* genes found in the genus Brassica encode TIR-NBS-LRR proteins [57].

## 4. Identification and Molecular Mechanism of Fusarium Wilt Resistance Genes

**Figure 3:**
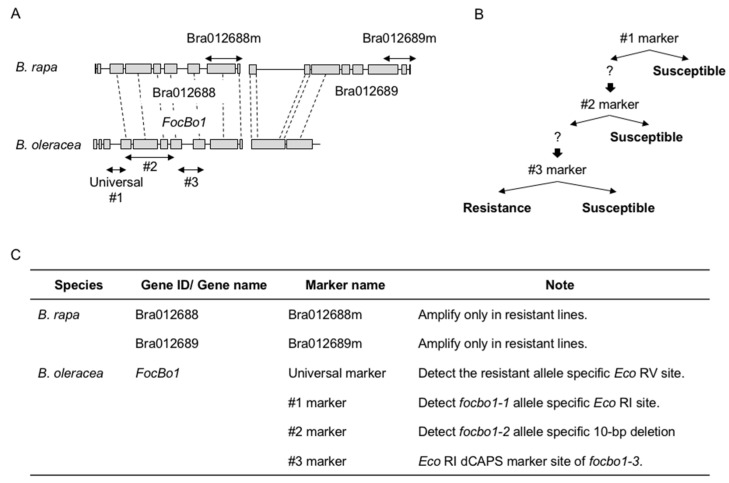
Schematic view of the alignment of resistance genes of Fusarium wilt disease. (**A**). DNA marker positions of resistance genes in *B. rapa* and *B. oleracea*. Arrows indicate marker positions. (**B**). Scheme of marker assisted selection in *B. oleracea*. (**C**). DNA marker list for marker assisted selection in *B. rapa* and *B. oleracea*.

Two types of resistance (Type A and Type B) in *B. oleracea* have been reported against Fusarium wilt [76]. Type A resistance is controlled by a single dominant gene and is stable at temperatures higher than 24 °C where Type B is polygenic and becomes unstable at temperatures above 24 °C [27,76,77]. Type A resistance is controlled by a single dominant gene against race 1 in *B. rapa* and *B. oleracea* and has been studied extensively in recent years (Figure 3, Table 4) [29,30,32,78,79,80].

In *B. rapa*, transcriptome analysis was performed using resistant and susceptible lines. The differentially expressed *R* genes were identified and seven dominant DNA markers at *R* genes were developed. Two dominant DNA markers on Bra012688 and Bra012689 were completely linked to the resistance phenotype by an inoculation test, indicating that these two genes are candidates for Fusarium wilt resistance genes in *B. rapa* (Figure 3, Table 4). These two genes encode TIR-NBS-LRR proteins [81]. Dominant DNA markers, Bra012688m and Bra012689m, were applied to Chinese cabbage inbred lines and confirmed close linkage to the Fusarium wilt resistant phenotype [82]. Furthermore, the transcriptome profiles following *Foc* inoculation between Fusarium wilt-resistant and -susceptible lines in *B. rapa* were compared and differentially expressed genes were identified [79]. These genes may be responsible for the resistance mechanism to *Foc* [79]. Differentially expressed genes between *B. rapa* and *Arabidopsis thaliana* after *Foc* inoculation at the same time point were compared and up-regulated genes related to defense response were identified [79], that may be candidates for conferring resistance against *Foc*.

Recently, Type A resistance has been mapped and molecular markers have been developed in *B. oleracea* [29,30,32]. The Fusarium wilt resistance gene, *FocBo1*, was mapped on chromosome 7 by both segregation testing and QTL analysis, and the closest simple sequence repeat (SSR) marker KBrS003O1N10 was developed [29]. One minor QTL was also detected on chromosome 4. In a previous study, the resistance gene on chromosome 6 of cabbage was linked to two insertion/deletion (InDel) markers: M10 and A1 [78]. Later, it was shown that the resistance of Fusarium wilt was controlled by a single dominant gene based on the segregation ratio of two populations (resistant inbred line, 99–77 and highly susceptible line, 99–91). Two *R* genes in the target region, re-Bol037156 and re-Bol0371578, were predicted as resistance genes, and re-Bol037156 gene, which encodes a putative TIR-NBS-LRR type R protein, has highly similar sequences among the resistant lines [31]. *FocBo1* locus was identified on chromosome 7 and this locus was fine-mapped by using 139 recombinant F_2_ plants derived from resistant cabbage (AnjuP01) and susceptible broccoli (GCP04) DH lines [30]. The *FocBo1* gene was shown by fine mapping to be an orthologous gene of Bra012688 in Chinese cabbage [30]. 

The proteome of xylem sap of the non-infected and *Foc* infected plants in both resistant and susceptible cabbage cultivars was also investigated using liquid chromatography-tandem mass spectrometry (LC-MS/MS) after the in-solution digestion of xylem sap proteins [83]. Twenty-five proteins in the infected xylem sap were found and ten of them were cysteine-containing secreted small proteins, suggesting that they are candidates for virulence and/or avirulence effectors. The transcriptome profiling of resistance to *Foc* in cabbage roots were also analyzed [26], where 885 differentially expressed genes (DEGs) were identified between infected and control samples at 4, 12, 24, and 48 h after inoculation. Some genes involved in Salicylic acid (SA)-dependent systemic acquired resistance (SAR), ethylene (ET)-, jasmonic acid (JA)-mediated, and the lignin biosynthesis pathways showed differential expression; the authors discussed the possibility that DEGs involved in these pathways may play important roles in resistance against *Foc* inoculation [26].

## 5. Resistant Breeding, Gene Accumulation, and MAS

MAS is an indirect selection process where a trait of interest is selected based on a marker (morphological, biochemical, or DNA/RNA variation) linked to that trait. Selecting individuals with disease resistance using MAS involves identifying a marker allele that is linked to disease resistance rather than to the level of disease resistance. There are several types of DNA markers that have been used to identify disease resistance genes [32,41,43,46,59,78,82,84].

The complexity of plant–pathogen interaction is a problematic in the case of CR breeding due to the appearance of multiple races of the pathogen [85]. Combinations of different *CR* genes exhibit higher resistance to the disease [62,67,86]. Though CR cultivars have been used widely for major production areas, field isolates of *P. brassicae* show variation, and different resistance sources from either *B. rapa* or *B. oleracea* vegetables were attained by *P. brassicae*. This suggests a serious risk that a resistance gene can be overcome by pathogen variants [3]. For example, seven CR canola cultivars were characterized for virulence in 106 *P. brassicae* population, and 61 of 106 *P. brassicae* population overcame the resistance in at least one of the seven CR cultivars [87]. There are many reports that *CR* genes show different reactions against the variable virulence of *P. brassicae* [34,36,44,48,61,62,63,66,74], but heterozygous *CR* loci are less resistant than the homozygous state [18]. *B. rapa* possesses several major *CR* loci (Table 1), which may confer differential (pathotype-specific) resistance to particular isolates of *P. brassicae*, and sometimes this may have a large effect on resistance [34,52,85,88]. The NARO Institute of Vegetable and Tea Science (NIVTS) has developed a high CR Chinese cabbage cultivar, ‘Akimeki’, by the accumulation of *Crr1*, *Crr2*, and *CRb* genes. It was proven that the accumulation of *CR* genes through MAS strengthened resistance and, consequently, it can be resistant to the multiple races of *P. brassicae* in *B. rapa*. Three *CR* genes, *CRa*, *CRk*, and *CRc*, were accumulated in Chinese cabbage through MAS [85] and the homozygous lines for the *CR* genes exhibited exceedingly high resistance against all six field isolates of *P. brassicae*. The effect of accumulation of different *CR* genes could be controlled by the dose-dependent accumulation of CR proteins [53,89]. In *B. oleracea,* resistance in genotypes has generally been identified less frequently than in the genotypes of *B. rapa* and the level of resistance is low [90]. This might be due to the polygenic nature of resistance in *B. oleracea* [67]. *B. oleracea* progeny were developed by accumulating major and minor QTLs to evaluate its effectiveness to the clubroot disease [64]. Three QTLs in the F_2_/F_3_ population from the cross between cabbage and kale line K269 were identified [62]. The accumulation of those three *CR* genes showed broad resistance to three isolates. It was observed that only one major QTL PbBo(Anju)1 showed moderate resistance, whereas three minor QTLs without the major one showed distinct susceptibility [64]. Later, it was proven that PbBo(Anju)1 and three minor QTLs PbBo(Anju)2, PbBo(Anju)4, and PbBo(GC)1 play a critical role in the acquisition of resistance to clubroot disease [67,86]. Here, *PbBo(Anju)1* plays a crucial role in the expression of clubroot resistance, and pyramiding minor *CR* genes are also essential for achieving higher resistance [67,86]. Their effectiveness was verified for controlling disease involving various isolates of *P. brassicae* [67]. Recently, two *CR* genes, *CRb* and *PbBa8.1*, were combined through MAS and CR homozygous lines in developed *B. napus*. The homozygous lines demonstrated a higher resistance than the heterozygous lines [91].

The Type A resistance to Fusarium wilt disease controlled by a single dominant gene has been successfully mapped and molecular markers have been developed: SSR marker KBrS003O1N10 [29], InDel markers M10 and A1 [78], Indel markers Bra012688m and Bra012689m [81,82], and DNA marker sets [30,80,84], which are used to generate a series of resistance cultivars (Figure 3).

Breeding cultivars that have resistance to both clubroot and Fusarium wilt is desired. However, inoculation tests against multiple pathogens or multiple races are difficult to perform on the same individual plant. Thus, DNA marker-based selection is useful for the identification of plants that have one Fusarium wilt resistance gene and multiple clubroot resistance genes. Furthermore, it is necessary to confirm whether these resistance genes are linked. In *B. rapa*, a Fusarium wilt resistance gene is located on chromosome 3, and *CRa/CRb*, *Crr3*, and *CRk* are located near this Fusarium wilt resistance gene. The *CRa/CRb* gene is the closest, being approximately 2 Mb in physical distance to the Fusarium wilt resistance gene (Figure 2). Since recombination between these two genes can occur [82], it is possible to inherit both resistance genes. In *B. oleracea*, a Fusarium wilt resistance gene is located on chromosome 7, and there is a minor QTL for clubroot resistance, PbBo(Anju)4, nearby this Fusarium wilt resistance gene. However, these loci are not completely linked to each other [81,83,84]. Therefore, it is possible to have both resistance genes. In *B. napus*, the association between susceptibility to Fusarium wilt and clubroot resistance against pathotype 3 was found, and these two resistance genes are located about 10 cM apart [92]. However, recombination between these two genes has been reported [92], suggesting that it is possible to inherit both resistance genes and identify them by DNA marker-based selection.

From the results from various researchers, it has been demonstrated that the DNA markers developed can select for the genes that are required for the acquisition of resistance, and these markers could be a powerful tool for resistance breeding in Brassica species. The novel breeding method developed can reinforce resistance by pyramiding *R* genes through MAS. For the genetic accumulation of *R* genes corresponding to wide pathogenicity, MAS is indispensable because it allows a precise identification of how many *R* genes are involved in a cultivar, and can monitor the accumulation of *R* genes in the progeny in the breeding program. To increase the durability of resistant cultivars to a broader spectrum of pathogen races, the combination of different *R* genes into a single line will be indispensable.

## 6. Conclusions

Brassica production is hampered by various diseases, especially clubroot and Fusarium wilt. Many types of *R* genes/QTLs have been identified in Brassica against the diseases and are being used for the improvement of resistance in cultivars. In case of clubroot disease, a total of 18 major *CR* loci have been identified in *B. rapa*, whereas only one major *CR* locus (*Rcr7*) and about 50 QTLs were detected in *B. oleracea*. Moreover, one locus (*Pb-Bn1*) on the A genome with more than 30 QTLs in *B. napus* and one locus (*Rcr6*) on the B genome in *B. nigra* were also identified. Several types of DNA markers that are linked with disease resistance allele have been developed, and they have been used for MAS. However, when there are several pathotypes, it is necessary to match effective *R* genes with a specific pathotypes and develop the DNA markers. The accumulation of *CR* genes corresponding to a wide pathogenicity will be important for breeding resistant cultivars.

A single type A dominant locus (*Foc-1*) was identified in *B. rapa* and *B. oleracea*, and several DNA markers have been developed.

*R* genes found from both diseases mostly encode a putative TIR-NBS-LRR. Understanding how plants cope with exposure to multiple pathogens such as *P. brassicae* and *Foc* will be important in breeding cultivars with multiple disease resistance.

## Figures and Tables

**Table 1:** CR loci reported on *B. rapa*.

QTLs	P/PR	Position	Linked Marker	Gene Source	References
*Crr1*	PR4	A08	BRMS-088	Turnip (G004-Siloga derived)	[34]
*Crr1a*	PR3,4	A08	BSA7	Turnip (G004-DH line)	[53]
*Crr1b*		A08	AT27		
*Crr2*	PR4	A01	BRMS-096	Turnip (G004-Siloga derived)	[34]
*Crr3*	PR3	A03	OPC11-2S	Turnip (Milan white)	[40,42]
*Crr4*	PR2,4	A06	WE24-1	Turnip (G004-Siloga derived)	[52]
*CrrA5*		A05	RAPD ^1^, SSR ^2^	Chinese cabbage (Inbreed line 20-2ccl)	[51]
*CRa*	PR2	A03	HC352b-SCAR ^3^	Chinese cabbage (DH line T136-8)	[39]
	PR2, P3			Chinese cabbage (CR Shinki)	[37,38]
*CRb*	PR2,4,8	A03	TCR09	Chinese cabbage (CR Shinki DH line, Akiriso)	[41]
	P3		KBrH059N21F	Chinese cabbage (CR Shinki)	[43]
	P3		B0902		[38,50]
	P4		KBrB085J21	Chinese cabbage (CR Shinki DH line)	[46]
*CRc*	PR2,4	A02	m6R	C9 (DH line of Debra)	[35]
*CRd*	PR4	A03		Chinese cabbage (Line 85–74)	[49]
*CRk*	PR2,4	A03	OPC11-2S	K 10 (DH line of CR Kanko)	[35]
*CRs*	P4	A08	SNP ^4^	Chinese cabbage (cv. Akimeki)	[54]
*Rcr1*	P3	A03	SSR ^2^	Flower Nabana (Pak choy)	[45]
	P2,5,6			Flower Nabana	[47]
*Rcr2*	P3	A03		Chinese cabbage (Jazz)	[48]
*Rcr4*	P2,3,5,6,8	A03	SNP ^4^	Chinese cabbage (T19)	[36]
*Rcr8*	P5X	A02			
*Rcr9*	P5X	A08			

P, pathotypes; PR, physiological race of *P. brassicae.* ^1^ RAPD-Random Amplification of Polymorphic DNA, ^2^ SSR-Simple Sequence Repeat, ^3^ SCAR-Sequence Characterized Amplified Region, ^4^ SNP-Single Nucleotide Polymorphism.

**Table 2:** CR loci reported on *B. oleracea*.

QTLs	P/PR	Position	Linked Marker	Gene Source	References
*Rcr7*	P3,5X	LG7		Cabbage cv. Tekila and Kilaherb	[57]
3 QTLs	PR7	LG1 LG4 LG9	14a 48 177b	Broccoli (CR-7)	[58]
2 QTLs	ECD 16/31/31	-	OPL6-780, OPB11-740, OPA18-14900, OPA4-700, OPE20-1250, OPA1-1880, OPA16-510	Kale (C10)	[59]
Pb-3 Pb-4	ECD 16/3/30	LG3 LG1	4NE11a 2NA8c	Cabbage (Bindsachsener)	[60]
1 QTL	PR2	LG3	WG6A1, WG1G5	Kale (K269)	[61]
QTL1 QTL3 QTL9	PR2	LG1 LG3 LG9	SCA02a2 SCB50b, SCB74c SOPT15a, SCA25	Kale (K269)	[62]
Pb-Bo1 Pb-Bo2 Pb-Bo3 Pb-Bo4 Pb-Bo5a Pb-Bo5b Pb-Bo8 Pb-Bo9a Pb-Bo9b	P1,2,4,7	LG1 LG2 LG3 LG4 LG5 LG5 LG8 LG9 LG9	Ae05.8800, T2 PBB38a, r10.1200 Ae15.100, RGA8.450 ELI3.983, aa9.983 PBB7b, ae05.135 ELI3.115, a18.1400 C01.980, t16.500 Aj16.570, W22B.400 A04.1900, ae03.136	Kale (C10)	[63]
Pb-Bo(Anju)1 Pb-Bo(Anju)2 Pb-Bo(Anju)3 Pb-Bo(Anju)4 Pb-Bo(GC)1	PR4	LG2 LG2 LG3 LG7 LG5	KBrHo59L13 CB10026 KBrB068C04 KBrB089H07 CB10065	Cabbage (cv. Anju)	[64,67]
2 QTLs	PR2	LG2		Cabbage (C1220)	[65]
1 QTL	PR9	LG3			
23 QTLs	PR4	-		Cabbage (GZ87)	[66]

P, pathotypes; PR, physiological race of *P. brassicae*; ECD, European Clubroot Differential set pathotype.

**Table 3:** CR loci reported on *B. napus* and *B. nigra*.

QTLs	PG/PR	Position	Process	Gene Source	References
*B. napus*					
*CR2a* *CR2b*	PR2	LG6 LG1	RFLP ^2^	Rutabage (cv. Wilhelmsburger)	[68]
*Pb-Bn1* 1 QTL 1 QTL	P4,7	A03 C02 C09	RAPD ^3^	Oilseed rape (cv. Darmor-bzh)	[69]
3 QTLs	SRSI	LG6 ^1^	AFLP ^4^, SSR ^5^	Canola (cv. Mendel)	[72]
19 QTLs	7 isolates with dissimilar P	A02, A03, A08, A09, C03, C05, C06, C09	AFLP ^4^, SSR ^5^	Oilseed rape (cv. Boohmerwaldkohl and ECD04)	[70]
5 QTLs	P3,5,6,8	A03	SSR ^5^/InDel ^6^	Canola (cv. Mendel)	[71]
1 QTL	P3	A03	PCR-based marker	Canola (DH line 12-3, ECD04 derived)	[73]
1 QTL	P2,3,5,6,8	A08	SSR ^5^	Rutabage (BF)	[74]
9 QTLs	P4	-		Oilseed rape (different accession)	[8]
2 QTLs	ECD 17/31/31	A02, A03	SNP ^7^	Oilseed rape	[75]
*B. nigra*					
*Rcr6*	P3	B03		Accession PI 219,576 (parental line)	[33]

P, pathotypes; PR, physiological race of *P. brassicae*; SRSI, Single Resting Spore Isolate of *P. brassicae*; ECD, European Clubroot Differential set pathotypes. ^1^ Dominant locus (with two recessive loci), ^2^ Restriction Fragment Length Polymorphism, ^3^ Random Amplification of Polymorphic DNA, ^4^ Amplified Fragment Length Polymorphism, ^5^ Simple Sequence Repeat, ^6^ Insertion-Deletion, ^7^ Single Nucleotide Polymorphism.

**Table 4:** Loci of resistance gene to *Foc* reported in Brassica species.

QTLs	Position	Linked Marker/Process	Gene Source	References
*B. rapa*				
*Foc-Br1a*	A03	Bra012688m	Chinese cabbage (F_2_ population)	[81]
*Foc-Br1b*		Bra012689m		
*B. oleracea*				
*FOC*	C06	InDel marker: M10 and A1	Cabbage (DH lines)	[78]
*FOC1* ^1^	C06	InDel marker: Bol037156 and Bol037158	Cabbage (DH line and F_2_ population)	[32]
*QTL1*	C04	SSR marker: KBrS003O1N10	Cabbage (AnjuP01): F_2_ population	[29]
*QTL2* (*Foc-Bo1*)^2^	C07			
*Foc-Bo1*^1^ (SDG)	C07	InDel marker: BoInd 2 and BoInd 11	Cabbage (AnjuP01): Recombinant F_2_ population	[30]

^1^ Single dominant gene, ^2^ major QTL.
